# A case report of a challenging diagnosis of biliary atresia in a patient receiving total parenteral nutrition

**DOI:** 10.1186/s12887-019-1446-2

**Published:** 2019-03-08

**Authors:** Atu Agawu, Andrew Wehrman, Jennifer Pogoriler, Natalie A. Terry, Henry C. Lin

**Affiliations:** 1 Division of General Pediatrics, Department of Emergency Medicine, Children’s Hospital of Philadelphia, 3401 Civic Center boulevard, 8W22, Philadelphia, PA 19104 USA; 2 Division of Gastroenterology, Hepatology, and Nutrition, The Children’s Hospital of Philadelphia, Philadelphia, PA USA; 3 Department of Pathology and Laboratory Medicine, The Children’s Hospital of Philadelphia, Philadelphia, PA USA

**Keywords:** Biliary atresia, Total parenteral nutrition, Cholestasis, Case report, Liver biopsy

## Abstract

**Background:**

Total parenteral nutrition (TPN) and biliary atresia (BA) are common causes of cholestasis in infancy. The diagnosis of BA is time sensitive due to an inverse correlation between age at intervention (hepatic portoenterostomy - HPE) and survival without liver transplantation. Clinical, laboratory, and histologic features of BA and parenteral nutrition associated cholestasis (PNAC) are similar, creating a diagnostic dilemma for cholestatic infants on parenteral nutrition. There is limited published information about the natural history of PNAC including time to resolution, or diagnostic tests that distinguish BA from other etiologies of cholestasis.

**Case presentation:**

We present a case of a child diagnosed with BA whose cholestasis began while receiving TPN. His clinical course was notable for transient resolution of his cholestasis after stopping parenteral nutrition and ultimate intraoperative diagnosis.

**Conclusions:**

Clinicians who care for patients who frequently receive TPN should be aware that clinical, laboratory, imaging, and biopsy findings can be similar between BA and PNAC.

## Background

Biliary Atresia (BA) is a cholangiopathy characterized by progressive sclerosing inflammation and fibrosis of the extrahepatic ducts [1]. BA presents in infancy with clinical symptoms of cholestasis (acholic stools, dark urine, jaundice). Diagnosis of BA remains challenging due to a frequent lack of pathognomonic findings, and its occurrence in a population with multiple potential etiologies of cholestasis [2, 3]. An additional complicating factor is that the prognosis for survival without liver transplant in BA is inversely correlated with the age at surgical management (hepatic portoenterostomy (HPE)), increasing the urgency for a timely diagnosis [4, 5]. As a result, clinicians are often in the position of needing to distinguish BA from non-BA causes of cholestasis in infancy. A relatively common etiology of cholestasis in hospitalized infants is parenteral nutrition associated cholestasis (PNAC), particularly in premature infants or others who cannot tolerate enteral nutrition [6]. PNAC is a diagnosis of exclusion and difficult to differentiate from BA on liver biopsy. Finally, the natural history of BA in premature infants (gestational age < 37 weeks) remains unclear, even though premature infants are often diagnosed at an older age than non-premature infants [7]. We present here a case of BA in a premature infant receiving TPN and the associated challenges in making the diagnosis and highlight the need for clinical judgement as well as new diagnostic testing tools.

## Case presentation

**Fig. 1:**
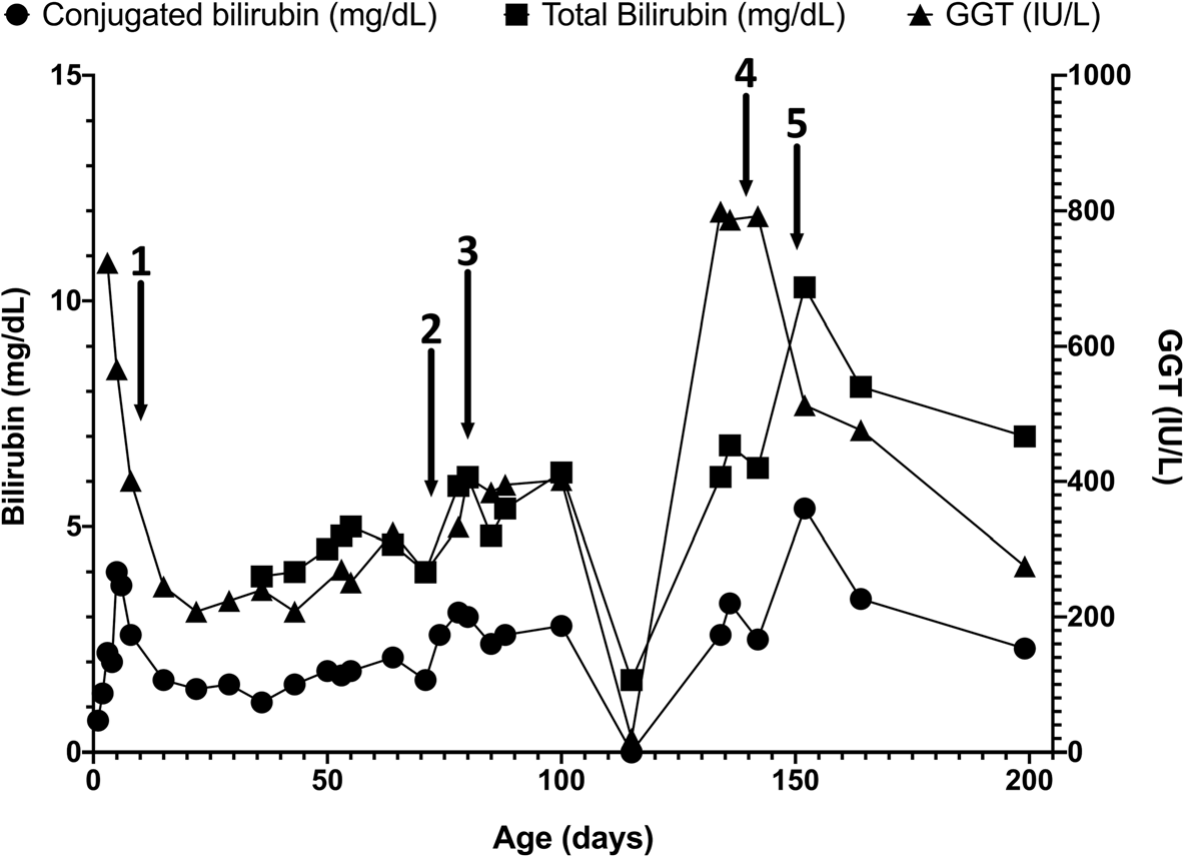
Conjugated bilirubin, total bilirubin, and GGT trends for patient. Arrows indicate significant clinical events as follows: 1. Enteral nutrition starts 2. TPN discontinued 3. Liver biopsy 1 4. Liver biopsy 2 5. HPE

**Fig. 2:**
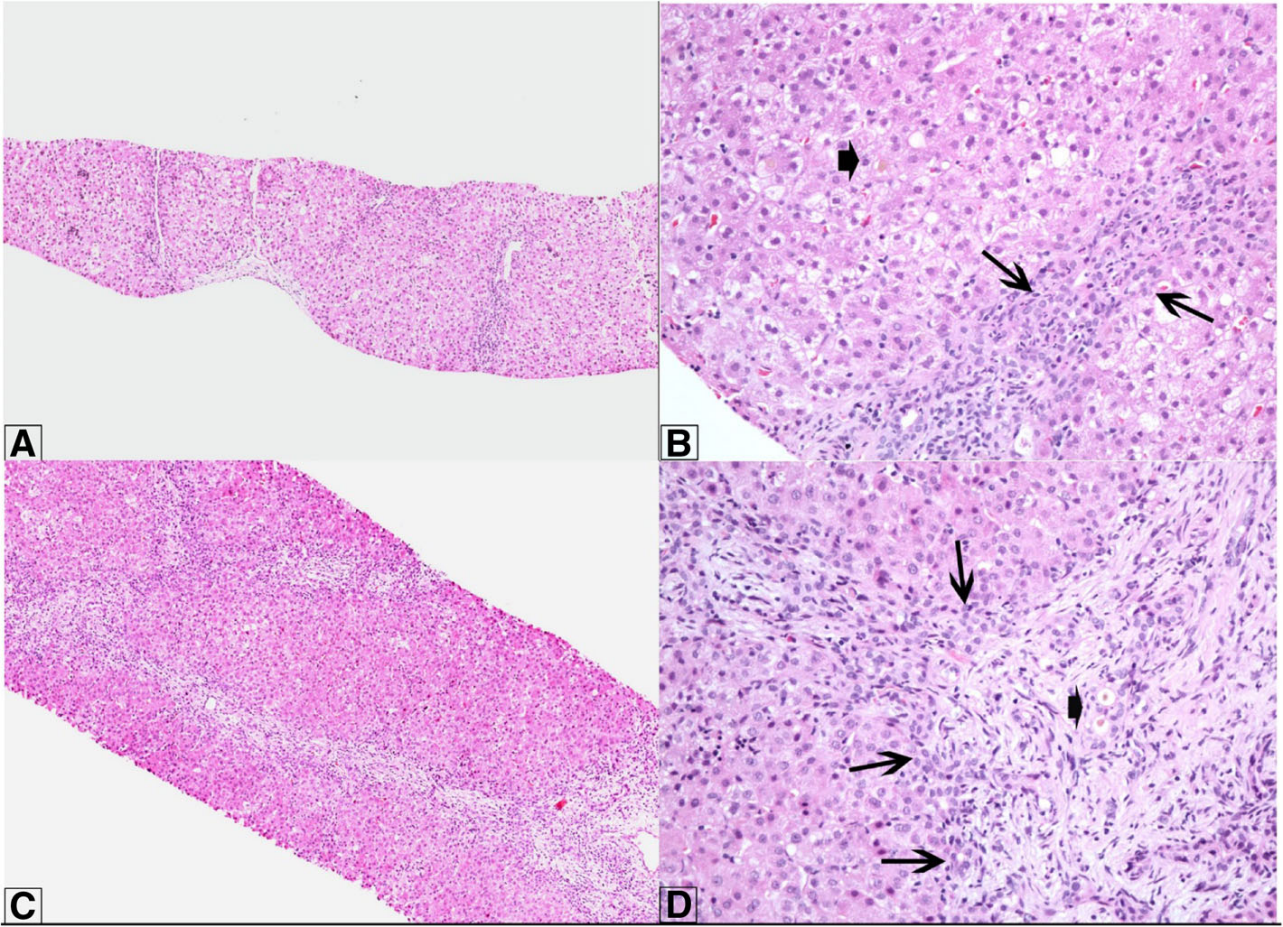
**a** and **b**: First biopsy (hematoxylin and eosin) at 81 days of life shows mild fibrosis (low power) and at high power bile ductular proliferation (long arrows) and canalicular cholestasis (short arrow) without bile duct plugging. Hepatocytes show mild steatosis. **c** and **d**: The second biopsy (hematoxylin and eosin) at 4 months of life shows more prominent bridging fibrosis (**c**) with bile ductular proliferation (long arrows) and new bile duct plugging (short arrow)

The patient is a 32-week twin gestation male infant admitted to a neonatal intensive care unit (NICU) at birth for bowel obstruction. Upper GI series and barium enema were performed on day of life (DOL) 1 and showed a lack of progression of contrast into the distal jejunum and a small colon, consistent with jejunal atresia. He subsequently underwent laparotomy which revealed a central segmental volvulus with ischemia. Approximately 35 cm of bowel were resected, an end ileostomy was created, and he was started on TPN. Enteral breast milk was started on DOL9, his ileostomy was re-anastomosed to his distal bowel on DOL 65, and he reached full feeds on DOL 73 at which point TPN was discontinued. Throughout his NICU course he maintained a conjugated hyperbilirubinemia (Fig. 1). On DOL 75 he had acholic stools and his conjugated bilirubin rose to 3.1 from 1.1 mg/dL (normal ≤0.3 mg/dL). Evaluation at the time included an abdominal ultrasound that did not visualize the gallbladder, and a non-excreting hepatobiliary iminodiacetic acid (HIDA) scan (after 22 h). He underwent liver biopsy on DOL 81 which showed minimal periportal fibrosis, mild bile ductular reaction, mild portal inflammation, as well as microvesicular steatosis and intrahepatic and canalicular cholestasis, findings which were consistent with effects of TPN but were not distinguishable from extrahepatic biliary atresia (Fig. 2a, b). His history of prolonged TPN exposure, absence of bile duct plugging and improving bilirubin (2.6 mg/dL from 3.1 mg/dL) suggested PNAC was the most likely etiology. In addition, screening for other causes of cholestasis (e.g. hypothyroidism, alpha-1- antitrypsin deficiency, galactosemia, cystic fibrosis, tyrosinemia, urinary tract infection, TORCH infections, etc.) was negative. He was monitored for several more days and subsequently discharged with close follow-up.

After discharge, the patient was closely followed and initially his conjugated hyperbilirubinemia improved (Fig. 1) and resolved on DOL 115. However, at his next check at DOL 134, his conjugated bilirubin rose to 2.6 mg/dL accompanied by elevations in his alanine aminotransferase (ALT), aspartate aminotransferase (AST), and gamma-glutamyl transferase (GGT), as well as persistent jaundice and scleral icterus. At this point, he underwent further evaluation. An abdominal ultrasound did not visualize the gallbladder and a HIDA scan did not excrete after 22 h, thus he underwent repeat liver biopsy which showed bridging portal fibrosis, bile duct proliferation and plugging, consistent with extrahepatic obstruction (Fig. 2c, d). Given these obstructive features, a cholangiogram was scheduled. During the procedure he was noted to have a small gallbladder remnant with no lumen large enough for a cholangiogram catheter, and a fibrotic extrahepatic biliary tree. He underwent HPE and was discharged on post-op day 6. After HPE, total bilirubin normalized and was < 2 mg/dL at 3 months post-HPE indicating success of the procedure [5].

## Discussion and conclusions

This prematurely-born infant presented with a cholestatic jaundice that transiently improved after discontinuation of parenteral nutrition but then recurred and was ultimately found to have BA. At the time of his initial liver biopsy, the patient had prolonged exposure to TPN and had only recently begun to tolerate full feeds. These factors, combined with an improving conjugated bilirubin, led to the decision that close clinical observation was appropriate since these findings were consistent with PNAC. Patients on TPN frequently have a conjugated hyperbilirubinemia and can have concurrent biochemical markers of liver injury as well as histologic changes ranging from steatosis to obstructive features with portal fibrosis and cirrhosis [8]. This patient’s conjugated hyperbilirubinemia was initially felt to be related to TPN initially, especially since work-up for additional causes of cholestasis was negative. In general, there is an expectation that after discontinuation of TPN, cholestasis should improve, although the time to resolution is not well characterized in the literature. Some patients have a rise in their conjugated bilirubin in the first month after TPN discontinuation prior to resolution. [9]

Although this patient initially followed an expected course for PNAC, his recurrent biochemical evidence of cholestasis and liver injury and continued jaundice prompted repeat evaluation. Particularly challenging in this case is the lack of histologic distinction between BA and PNAC on liver biopsy [10]. Since BA and PNAC share histologic findings of obstructive cholestasis, additional tests are needed to help distinguish the diagnoses. HIDA scan is commonly used to help distinguish BA from non-BA etiologies and has been shown to have high sensitivity (98.7%) but low specificity (70.4%) [11], meaning that 30% of patients with non-excreting HIDA scans will ultimately have a normal extrahepatic biliary tree. Both PNAC and BA cause obstructive cholestasis, and recent studies have shown no pathologic basis for distinguishing the two diseases [10, 12]. Given the limits of imaging, biomarker, and pathology studies, intraoperative cholangiogram with the option for HPE remains the gold standard for the diagnosis of biliary atresia, especially given the time-sensitive nature of diagnosis and intervention [4], and was the modality that provided the definitive diagnosis in this case. A question that naturally emerges for the patient in question was whether he should have undergone interoperative cholangiogram with his initial cholestasis evaluation. Although it seems that an earlier cholangiogram could have identified his BA sooner, it is also possible that the full obliteration of his biliary tree had not yet occurred, and he could have had a falsely negative intraoperative cholangiogram. A second question that emerges is when and how to evaluate patients with long-term TPN requirements for biliary atresia, which was especially challenging in our patient. Finally, it remains unclear from the literature whether the timeline for premature infants should include their corrected or chronological age [7].

This case highlights the ongoing barriers that exist for timely diagnosis of biliary atresia despite the availability of multiple diagnostic tests. Clinicians who care for patients who may have multifactorial causes of their cholestasis should be aware that liver biopsy by itself cannot distinguish biliary atresia from PNAC and know the appropriate places to refer patients who may need invasive diagnostic testing (e.g. intraoperative cholangiogram). This study also reinforces the need for more non-invasive diagnostic tests that are specific to biliary atresia.

## Abbreviations

ALT: alanine aminotransferase
AST: aspartate aminotransferase
BA: Biliary Atresia
DOL: Days of Life
GGT: Gamma-glutamyl transferase
HIDA: hepatobiliary iminodiacetic acid
HPE: hepatic portoenterostomy
NICU: Neonatal intensive care unit
PNAC: Parenteral Nutrition Associated Cholestasis
TPN: Total Parenteral Nutrition
